# Miscellaneous Medical Intelligence

**Published:** 1852-01

**Authors:** 


### MISCELLANEOUS MEDICAL INTELLIGENCE.

The N. O. Med. and Surg. Journal, takes from L'Union Medicale for Aug., 1851, notice of a case of suicide effected by Chloroform. Dr. Rayer, Physician *en chief* of the Royal Hospital of Vienna, terminated his earthly career, in the midst of his colleagues, by taking Chloroform. Up to the time of his death, his health was good and his intellect sound. He was found dead in his chamber with his nose and mouth plunged into an ætherization sac filled with Chloroform, which he had taken the precaution to fix with plaster of diachylon about his face.

Kate Dresser, 39 years old, of Schuylkill Co., Penn., has had more children than most women. The first child was born in 1829, and the last in February, 1851. She has had twins five times, and in February, 1848, had four children at one birth! making twenty-one children in twenty-one years, and six children in the space of eighteen months! The four children at a birth were apparently healthy and well formed.

One lived about four weeks, another eleven months, the third a little over a year, and the fourth, a fine boy, is still living. There are now twelve of the whole number living, seven boys and five girls!

Prof. Johnson, of Va., says two parts of vinegar to one of salt, in doses of a table-spoonfull every three hours, has been found useful in obstinate diarrhœas.

Our Ohio friends are providing themselves with splendid Medical College buildings, one at Columbus and one at Cincinnati being nearly completed.

An ounce of quinine was given to a negro at Hillsboro, N. C., in twelve hours for congestive fever. He recovered.

